# A phase 1 study of the BCL2-targeted deoxyribonucleic acid inhibitor (DNAi) PNT2258 in patients with advanced solid tumors

**DOI:** 10.1007/s00280-013-2361-0

**Published:** 2013-12-03

**Authors:** Anthony W. Tolcher, Wendi V. Rodrigueza, Drew W. Rasco, Amita Patnaik, Kyriakos P. Papadopoulos, Alex Amaya, Timothy D. Moore, Shari K. Gaylor, Charles L. Bisgaier, Mina P. Sooch, Michael J. Woolliscroft, Richard A. Messmann

**Affiliations:** 1 South Texas Accelerated Research Therapeutics (START), LLC, San Antonio, TX USA; 2 ProNAi Therapeutics, Inc., 46701 Commerce Center Dr., Plymouth, MI 48170 USA; 3 Mid-Ohio Oncology Hematology, Inc., Columbus, OH USA

**Keywords:** PNT2258, Phase I, DNAi, Deoxyribonucleic acid inhibitor, BCL2, Liposome

## Abstract

**Purpose:**

Maximum tolerated dose, safety, pharmacokinetics, and pharmacodynamics were assessed in this phase 1 study of PNT2258, a BCL-2-targeted liposomal formulation of a 24-base DNA oligonucleotide called PNT100.

**Methods:**

Patients with malignant solid tumors were assigned sequentially to one of ten dose-escalation cohorts of PNT2258 at 1, 2, 4, 8, 16, 32, 64, 85, 113, and 150 mg/m^2^ administered intravenously on days 1 through 5 of each 21-day cycle. Pharmacokinetics were determined on days 1 and 5 of the first cycle. Lymphocyte and platelets concentrations were measured for evidence of BCL2-targeted effect. CT scans were used to identify radiologic evidence of anti-tumor effect.

**Results:**

Twenty-two subjects received PNT2258, and the maximum tolerated dose for PNT2258 was not reached. Doses at or above 32 mg/m^2^ resulted in exposure to PNT2258 above the exposure level required for anti-tumor activity in preclinical xenograft testing of 22,377 ng h/ml (PK analysis 2012). Fatigue was the most commonly reported adverse event. Dose-limiting toxicity, manifesting as a transient increase in aspartate aminotransferase, occurred at 150 mg/m^2^, the highest dose tested. Four subjects, two each with diagnosis of non-small-cell lung cancer and sarcoma, treated at doses of 64 mg/m^2^ or higher, remained on study for 5–8 cycles.

**Conclusions:**

PNT2258 was safe and well tolerated at the doses tested up to 150 mg/m^2^. Exposure to PNT2258 resulted in clinically manageable decreases in lymphocyte and platelet concentrations.

## Introduction

BCL2 and its related family of proteins play a key and central role during embryogenesis, homeostasis, as guardians of mitochondrial function, and in the regulation of apoptosis and autophagy subsequent to cellular injury or stress [1–3]. The derangement of BCL2 regulated control mechanisms is a defining characteristic of certain malignancies including subsets of non-Hodgkin’s lymphoma (NHL) and chronic lymphocytic leukemia [4].

This phase 1 study is the first-in-human assessment of PNT2258. The primary endpoints of the study included identification of the maximum tolerated dose of PNT2258 and characterization of the safety and toxicity profile of PNT2258 when administered to patients with advanced solid tumors. Secondary objectives included characterization of the PNT2258 pharmacokinetic profile and identification of any anti-tumor effect that may occur. The exploratory objective for the study included analysis of peripheral blood mononuclear cells (PBMCs), lymphocyte and platelet concentrations, plasma and serum samples for evidence of BCL2-mediated effect.

### PNT2258

**Fig. 1:**
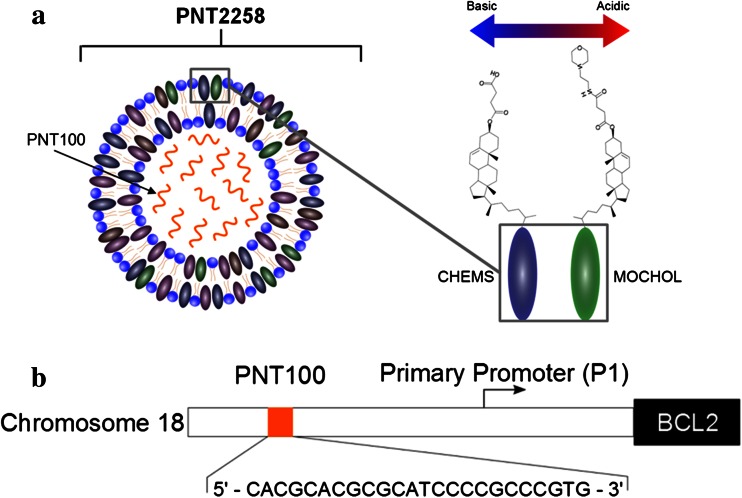
**a** A representation of the PNT2258 molecule. The PNT100 oligonucleotide is encapsulated in liposomes of MOCHOL/CHEMS/DOPE/POPC, particle size ~130 nm. PNT2258 has an overall pKa of ~6.5, containing pH responsive lipids that are cationic during manufacturing (in order to attract the negatively charged PNT100 oligonucleotide) and anionic during systemic circulation. Charge and lipid components are similar to circulating lipoproteins The pH “tunability” is designed to enhance endosomal escape following cellular uptake. **b** Diagrammatic representation of the DNA “target” for PNT100 binding located on chromosome 18. The PNT100 oligonucleotide sequence is designed to hybridize (i.e., bind) to a region 5′ upstream of the BCL2 gene start site

PNT2258, the anti-BCL2 experimental therapeutic used in this study, consists of a protective liposomal formulation composed of four lipids encapsulating a 24-base, chemically unmodified DNA oligonucleotide called PNT100. A representation of the PNT2258 molecule is illustrated in Fig. 1a. The lipid components of this PNT2258 nanoparticle are comprised of 1-palmitoyl-2-oleoyl-sn-glycero-3-phosphocholine (POPC), 1,2-dioleoyl-sn-glycero-3-phosphoethanolamine (DOPE), cholesteryl hemisuccinate (CHEMS), and cholesteryl-4-([2-(4-morpholinyl)ethyl]amino)-4-oxoburanoate (MOCHOL) [5]. These nanoparticles are anionic at physiological pH, and their specific lipid ratio imparts a “pH-tunable” character and a charge to the liposomes, which changes depending upon the surrounding pH of the microenvironment to facilitate movement across physiologic membranes [6, 7]. The PNT2258 nanoparticle is sized to avoid extensive hepatic sequestration, with an average diameter of approximately 130 nm, facilitating systemic distribution and serum stability after intravenous injection [8]. These specialized liposomes are also known by the registered trade name as SMARTICLES^®^.

Preclinical studies of injected PNT2258 demonstrated systemic biodistribution of the active pharmaceutical ingredient (PNT100) with accumulation into tissues including xenograft tumors [9].

### PNT100

PNT100 is a single-stranded DNA 24-base oligonucleotide encapsulated in the aqueous core within the liposomal nanoparticle. PNT100 is a solid-phase on-column chemically synthesized oligonucleotide that is cleaved from the column solid support, followed by ion-exchange (IEX) purification, ultra-filtration/diafiltration (UF/DF), and concentration with subsequent freeze-drying. PNT100 has the sequence, 5′-CACGCACGCGCATCCCCGCCCGTG-3′. PNT100 is specific to regulation of the BCL2 gene and not designed to hybridize to the regulatory areas of other BCL2 protein family members such as Bcl-xL, MCL-1, or Bcl-w. HeLa cells exposed to Cy5.5-fluorescently labeled liposomal encapsulated PNT100 showed fluorescence concentrated in nuclei with diffuse perinuclear distribution [10].

The PNT100 sequence is designed to hybridize with genomic sequences that reside within 5′-untranscribed regulatory regions of the BCL2 gene to block transcription of BCL2 through a mechanism called DNA interference (DNAi) (Fig. 1b). In cancerous cells and in contrast to normal cells, these upstream regulatory regions are susceptible to hybridization by having an open chromatin state that occurs during oncogene up-regulation [11]. Additionally, the PNT100-targeted area of the genome is included in translocation *t*(14,18) [12]. The interaction of the PNT100 DNAi oligonucleotide with the targeted non-coding, non-transcribed genomic DNA was designed to block transcription and silence BCL2 transcription, prompting initiation of the cell death pathway and pro-apoptotic events that lead to cancer cell death.

### Preclinical studies

Liposomal formulations of PNT100 given IV have demonstrated dose-dependent, single-agent efficacy, including complete and partial tumor regressions and extended tumor growth delays in a WSU-DLCL2 murine xenograft model of human NHL [13]. In addition, synergistic in vivo anti-tumor effect was noted with liposomal PNT100 in combination with rituximab or docetaxel in xenograft models of human Burkitt’s lymphoma and melanoma, respectively [8].

Anti-tumor effects in mice bearing WSU-DLCL2 NHL xenograft tumors were only observed when the animals received liposomal PNT100, but not a liposomal formulation of either a scrambled 24-base control or a 2-base mismatch oligonucleotide (i.e., differing in sequence from PNT100 at 2-bases). These results suggest that a sequence-specific genomic interaction contributed to the DNAi mechanism of action.

The preclinical pharmacokinetic characterization of PNT2258 consisted of analyzing PNT100 concentration, the active oligonucleotide ingredient in rodent and primate plasma obtained from a toxicity and safety pharmacology study. In this study, PNT2258 was administered for 5 consecutive days during two cycles separated by 2 weeks [14, 15]. PNT100 was detected by a hybridization-ligation assay after detergent disruption of the PNT2258-containing plasma samples. The probe used in the assay was specific for full-length human PNT100. The non-competitive hybridization and ligation assay (by ELISA) was validated in monkey plasma and cross-validated in human plasma (including interference for hemolysis) and was used in this study [16]. No free PNT100 was detected in the samples prior to detergent disruption of the lipid nanoparticle. The data obtained from measurement of monkey plasma PNT100 concentration demonstrated greater than dose-proportional exposure and an apparent multi-compartmental and saturable clearance process. The clearance of the PNT2258 liposomes from circulation was presumed to be mediated by mononuclear phagocytes residing primarily in the spleen and liver (Kupffer cells) as visualized during histopathology in the toxicology studies in both cynomolgus monkeys and rats [14, 15, 17]. The tissue distribution is consistent with other known liposomal formulations [18].

### Human study dose selection

In addition to the cynomolgus monkeys, rats were used as the second species for PNT2258 preclinical toxicity studies. A lowest no observed-adverse effect level (NOAEL) for PNT2258 was defined at 15 mg/kg/day in rats and 16 mg/kg/day in monkeys. The IV starting dose of PNT2258 in the clinical phase 1 study was 1.0 mg/m^2^, representing 1/6th the NOAEL in rats based on a corresponding human body surface area of 1.6 m^2^.

## Materials and methods

### Patient selection

The study population included only patients with metastatic solid tumors that had exhausted standard therapeutic options; were age ≥18 years; and had Eastern Cooperative Oncology Group performance status ≤2 with adequate bone marrow, hepatic, and renal function. As this was a pilot dose finding and toxicity-characterization phase 1 study, pre-identification of patients on the basis of their existing BCL2 tumor status was not a requirement for study participation. All subjects provided written informed consent.

The trial was conducted in accordance with precepts established by the Helsinki Declaration. The institutional review board approved the protocol, and the study was registered at ClinicalTrials.gov and given the identifier number of NCT01191775 [19].

### Study design and treatment schema

PNT2258 was supplied in 20 mL vials (with a target concentration of 2.5 mg/mL PNT100) and kept frozen (−20 ± 5 °C) during storage. PNT2258 was allowed to thaw at room temperature and subsequently diluted in 200 mL of 0.9 % sodium chloride in preparation for IV administration. Patients received PNT2258 as a 2-hour IV infusion, once daily on days 1–5 of a 21-day cycle. Individual patient doses were calculated based upon body surface area (with capping at a maximum of 2 m^2^) obtained prior to dosing on C1D1 with an allowed adjustment in dose for ≥10 % change in body weight during the study.

The initial dose level (i.e., cohort 1) was 1 mg/m^2^. Doses 1 through 64 mg/m^2^ allowed 1 patient per cohort until the occurrence of a dose-limiting toxicity (DLT), triggering cohort expansion to 3 patients. Doses 85 through 150 mg/m^2^ required 3 patients per cohort until the occurrence of a DLT. Assuming 0–1 DLTs at any dose level, patients were treated at the next higher dose level. If at any time 2 or more patients developed DLTs within a dose level, the level was considered too toxic, and the next lowest dose level was considered the recommended phase 2 dose.

All toxicities, regardless of attribution, were considered in determining whether a DLT had occurred at any particular dose and DLT was defined as any of the following events experienced during cycle 1: grade 4 neutropenia of >5 days duration or grade 3 or greater febrile neutropenia of any duration; grade 4 thrombocytopenia; any grade 3 or greater non-hematologic toxicity (except alopecia, nausea/vomiting well controlled with antiemetics, and laboratory abnormalities felt to be clinically insignificant or that were elevated at baseline); any toxicity resulting in a treatment delay of >2 weeks; an acute infusion reaction that did not resolve to baseline or ≤grade 1 after infusion interruption and resumption at a slower rate; a 2-grade increase in AST (SGOT)/ALT (SGPT) for patients with baseline grade 1 or 2 abnormalities. The maximum tolerable dose (MTD) was defined prospectively as the dose level below the dose where ≥2 of 6 patients experienced DLT.

Sequential cohort doses were doubled (i.e., 1, 2, 4, 8, 16 and 32 mg/m^2^) until the 64 mg/m^2^ dose level; thereafter, dose escalation proceeded in 33 % dose increments from 85 to 113 mg/m^2^ and to a final dose of 150 mg/m^2^.

### Baseline and treatment assessments

Baseline and on study assessments included medical history, physical examination, vital signs, ECOG performance status, ECG, radiographic tumor measurement, hematology, clinical chemistry, urinalysis. Hematologic and non-hematologic data were assessed according to the Common Terminology Criteria for Adverse Events (CTCAE) rating scale, version 3.0 [20]. Tumor response was assessed using Response Evaluation Criteria in Solid Tumors (RECIST) 1.0 guidelines [21].

### Pharmacokinetic and pharmacodynamic analyses

The concentration of encapsulated PNT100 was measured at predetermined sampling time points using the hybridization-ligation assay by Charles River Labs (Montreal, Canada). Assay validation demonstrated specificity, precision, accuracy, inter-assay variation, long-term stability, and no interference with 5 % (v/v) hemolysis in plasma with a lower limit of quantitation (LLOQ) of 3 ng/mL [16].

A nonlinear mixed effect modeling technique (NONMEM, version VI) was used to analyze plasma PNT100 data (log-transformed), allowing estimation of mean PK parameter values and inter-individual and intra-individual variability. Model parameters were estimated using the first-order conditional estimation method with interaction. The inter-individual and intra-individual variability were coded as an exponential and a proportional relationship, respectively. Three and 4 compartmental PK models with elimination from the peripheral compartment and a similar elimination rate constant were fitted to the PK data.

PNT100 plasma concentration data were also analyzed using a standard non-compartmental method to derive PK parameters for PNT100: maximum plasma concentration (*C*_max_), area under the plasma concentration versus time curve (AUC), and serum half-life (*t*_1/2_).

Lymphocyte and platelets concentrations were determined from patient samples obtained at prespecified time points throughout the study and analyzed per standard clinical criteria.

### Statistical analysis

Patient demographics, adverse events, clinical laboratory evaluations, and vital signs were summarized descriptively. Quantitative laboratory measurements were categorized according to normal reference ranges.

All patients that received PNT2258 were considered evaluable for the safety analysis. Patients that received at least 80 % of the planned dose in cycle 1 were considered evaluable for the purpose of dose escalation.

The efficacy-evaluable population included all patients with measurable disease that received at least one full cycle of PNT2258 and had at least one post-treatment response assessment or discontinued before having a response assessment due to rapid disease progression or death. Descriptive statistics were used for plasma concentration data.

## Results

### Patients

Twenty-two patients were accrued and received PNT2258 at START—South Texas Accelerated Research Therapeutics, San Antonio, Texas, between September 2010 and January 2012. Table 1 summarizes patient demographics, disease characteristics, and prior treatment.

**Table 1:** Patient demographics and disease characteristics

Parameter	*n* = 22
Median age (range)	63 (30–91)
Gender, *n* (%)	
Male	12 (55)
Female	10 (45)
Origin	
Caucasian	18 (82)
African	2 (9)
Hispanic	2 (9)
ECOG performance scale, *n* (%)	
0	3 (14)
1	16 (72)
2	3 (14)
Disease stage at entry, *n* (%)	
Stage IV	22 (100)
Pathologic diagnosis	
Pancreatic cancer	5
Colon adenocarcinoma	5
Sarcoma	3
Prostate, adenocarcinoma	2
Lung, non-small-cell carcinoma	2
Breast, adenocarcinoma	1
Endometrial	1
Head and neck carcinoma	1
Hepatocellular carcinoma	1
Neuroendocrine tumor	1
Prior treatment	
Systemic therapy	22 (100)
Surgery	17 (77)
Radiotherapy	8 (36)

*ECOG* Eastern Cooperative Oncology Group

### Treatment

Patients received PNT2258 at doses ranging from 1 to 150 mg/m^2^, constituting over 300 doses and sixty 21-day cycles. Over all dose levels, patients received a median of 2 completed cycles, with a range of 0–8 cycles. The most common reasons for study discontinuation were progressive disease or symptomatic deterioration (19 patients, 86 %). Two patients (9 %) discontinued therapy due to adverse event, and 1 patient (4 %) withdrew informed consent. Of the 335 planned doses of PNT2258, 314 were administered as scheduled. No patients were dose reduced for toxicity.

PNT2258 was well tolerated at dose levels 1 through 64 mg/m^2^. A single patient manifested a grade 3 DLT of back/flank pain while receiving the infusion at the 85 mg/m^2^ dose level, triggering expansion of the cohort to six subjects. After the occurrence of the DLT and at the discretion of the treating physician, subsequent patients could receive premedication prior to infusion with dexamethasone 10 mg, diphenhydramine 50 mg, and ranitidine 50 mg IV, either alone or in combination, on day 1 of each cycle as prophylaxis for back/flank pain. This intervention limited additional occurrences of flank/back pain. No additional DLTs occurred at the 85 mg/m^2^ dose level or at 113 mg/m^2^. One DLT occurred at 150 mg/m^2^ manifesting as a grade 3 increase in AST that resulted in expansion of the cohort. No additional cycle 1 DLTs were noted at the 150 mg/m^2^ dose level. However, a patient developed a grade 4 thrombocytopenia within 30 days of study participation at the 150 mg/m^2^ dose level.

Table 2 summarizes the drug-related toxicities at all dose levels. Across all dose levels and regardless of attribution, a total of 79 adverse events and 6 serious adverse events (see section below) were reported. The most common AEs were fatigue (8 events in 7 subjects; 8/79, 10.1 %; grade range 1–2) and infusion reaction manifesting as back or flank pain (6 events in 4 subjects; 6/79; 7.6 %; grade range 2–3). The increase in aspartate aminotransferase at the 150 mg/m^2^ dose level was observed in a patient with metastatic disease to the liver and elevated levels resolved spontaneously within 48 h. One patient died, as a result of disease progression, within 30 days of study participation.

**Table 2:** Treated patients (*n* = 22) with CTCAE 3.0 drug-related toxicities^a^

Dose level and no. of pts.	1 mg/m^2^ *n* = 1	2 mg/m^2^ *n* = 1	4 mg/m^2^ n = 1	16 mg/m^2^ *n* = 1	64 mg/m^2^ *n* = 1	85 mg/m^2^ *n* = 6		113 mg/m^2^ *n* = 3	150 mg/m^2^ *n* = 6	Total no. (%)
Adverse event	G1/2 *n* (%)	G1/2 *n* (%)	G1/2 *n* (%)	G1/2 *n* (%)	G1/2 *n* (%)	G1/2 *n* (%)	G3 *n* (%)	G1/2 *n* (%)	G1/2 *n* (%)	
Infusion reaction (i.e., back/flank pain)					1 (4)	1 (4)	1 (4)	2 (9)	1 (4)	6 (27)
Nausea		1 (4)	1 (4)	1 (4)					1 (4)	4 (18)
Vomiting		1 (4)				1 (4)				2 (9)
Diarrhea	1 (4)								1 (4)	2 (9)
Increased AST									1 (4)	1 (4)
Thrombocytopenia									1 (4)	1 (4)

*Gr* grade, *pts* patients, *no* number, *mg* milligrams, *m* meter

^a^Listed adverse events are those assessed by the investigators as possible, probably or definitely related to study drug collected from the first dose of study drug until 30 days after discontinuation from the study. Patients are counted only once per event, with the highest experienced grade noted. No drug-related toxicity was reported in 2 or more patients at the 8 and 32 mg/m^2^ dose levels. Only toxicities reported in 2 or more patients over all cycles are reported with the exceptions stated below. A single grade 4 thrombocytopenia was noted during cycle 2 in the highest dose tested (i.e., 150 mg/m^2^). A single grade 3 toxicity of increased AST occurred at the 150 mg/m^2^ dose level

### Efficacy

**Fig. 2:**
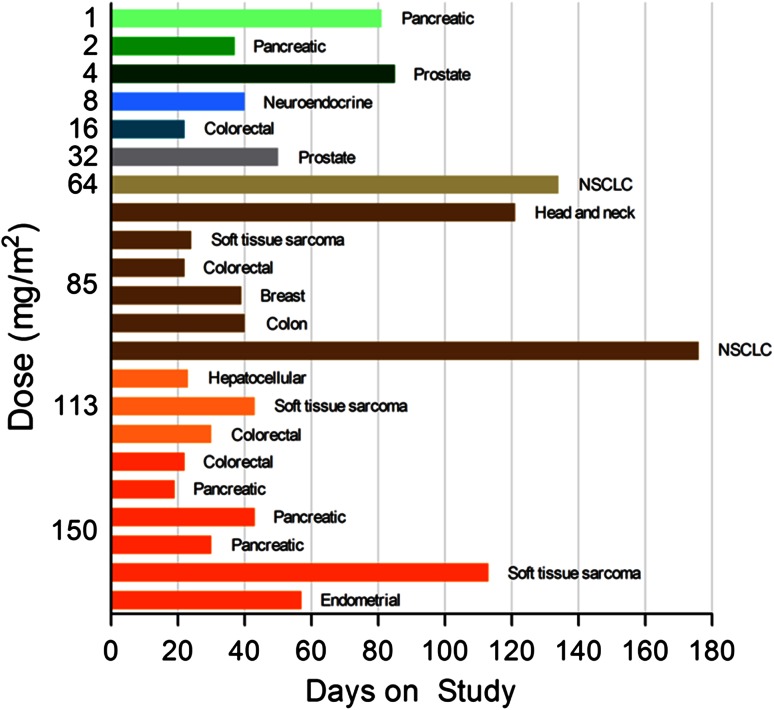
Days on study for each subject as a function of dose of PNT2258 administered. Patient cancer diagnosis is shown to the right of the *bars*

Of the 22 patients on study, 27 % (6 of 22) had stable disease at the time of the end-of-cycle 2 CT scan. Four patients, 2 each with a diagnosis of NSCLC at the 64 and 85 mg/m^2^ dose levels and sarcoma at the 85 and 150 mg/m^2^ dose levels, remained on study for >4 cycles (range 5–8 cycles). See Fig. 2.

### Pharmacokinetics

Plasma was obtained to evaluate the pharmacokinetics of PNT2258 (Table 3). Profiles were generated from C1D1 and C1D5 dosing to calculate *C*_max_, *t*_1/2_, and AUC_∞_. *C*_max_ values for C1D1 and C1D5 were consistent for dose cohorts with higher doses exhibiting an increase between C1D1 *C*_max_ versus C1D5 *C*_max_ and *C*_max_ values in patients achieved at the higher dose levels approached those achieved in cynomolgus monkeys on C1D1.

**Table 3:** PNT2258 plasma pharmacokinetic parameters following day 1 and 5 dosing (2-hour IV infusion)

Dose mg/m^2^	Day 1				Day 5			
	No. of patients	*C*_max_ (ng/mL)	*T*_1/2_ (h)	AUC_∞_ (ng h/mL)	No. of patients	*C*_max_ (ng/mL)	*T*_1/2_ (h)	AUC_∞_ (ng h/mL)
1	1	15.87	334.4	1,559	1	14.09	–	–
2	1	28.02	–	–	1	32.24	47.1	1,313
4	1	39.55	–	–	1	98.30	82.2	7,404
8	1	132.26	74.1	1,888	1	97.10	46.0	2,550
16	1	317.33	–	–	1	522.36	45.1	6,731
32	1	894.32	21.9	29,279	1	2,477.54	58.5	131,853
64	1	5,306.63	10.9	57,543	1	8,141.03	35.1	193,421
85	6	7,895 (35)	27.8 (73)	146,685 (67)	6	12,311 (54)	50.3 (27)	607,765 (75)
113	3	29,222 (40)	10.2 (42)	204,148 (61)	3	42,125 (91)	32.1 (26)	849,077 (123)
150	6	28,943 (39)	8.7 (57)	203,879 (49)	6	35,926 (43)	26.8 (47)	1,151,961 (60)

Numbers in parentheses indicate coefficient of variation (%). – indicates data not available

AUC_∞_, area under the concentration–time curve from zero to infinity; C_max_, peak (maximum) plasma concentration; t_1/2_, half-life; IV, intravenous

Serum half-life (*t*_1/2_) could not be consistently calculated for C1D1 for dose cohorts 1–5, owing to rapid clearance kinetics of PNT2258 at these lower doses. *t*_1/2_ at the dose levels ≥32 mg/m^2^ ranged from 8.7 to 58.5 h. An increase in *t*_1/2_ over C1D1 was noted for C1D5. These results are consistent with the expected prolonged clearance kinetics of PNT2258 due to saturation of the reticuloendothelial system (RES) clearance mechanisms and are similar to those noted in the toxicology studies [14, 15, 17].

The results showed consistent *T*_max_ of about 1.96 h, corresponding to the end of infusion sampling (data not shown).

A dose-dependent increase in AUC was noted with exposure levels in patients. In addition, a similar trend of increasing AUC was noted between C1D1 and C1D5 with AUCs in the latter sampling approaching those observed at the highest dose level tested in preclinical toxicology studies [22]. A less than dose-proportional increase was noted at the higher dose cohorts and may be indicative of achieving the maximum exposure of PNT2258.

The exposure levels (AUCs) in patients at doses at or above 32 mg/m^2^ (range 29,279–1,151,961 ng h/ml) exceeded levels at which preclinical anti-tumor effects were observed in xenograft studies (22,377 ng h/ml) [1, 22].

### Pharmacodynamics

**Fig. 3:**
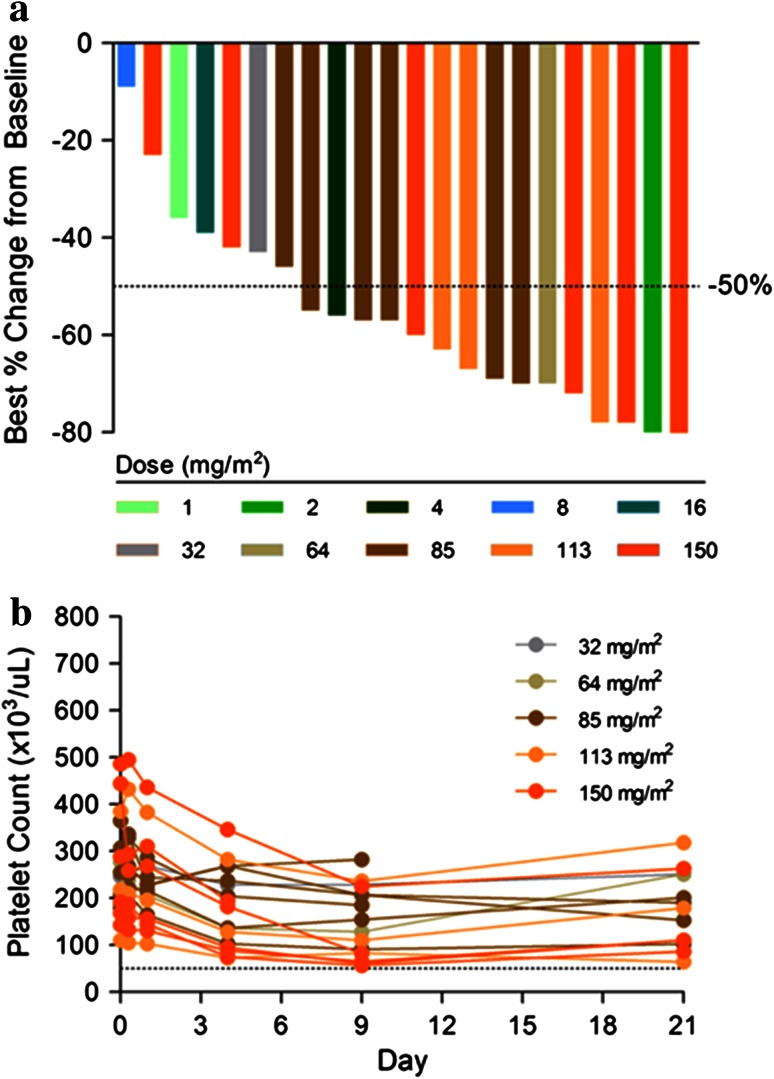
**a** First-cycle lymphocyte nadir (i.e., percent maximum decline from baseline) as a function of dose. **b** Cycle 1 platelet levels

Figure 3a illustrates lymphocyte concentrations in patients receiving PNT2258. Maximum decrease and leveling of lymphocytes concentrations occurred within hours of the onset of PNT2258 infusion.

As shown in Fig. 3b, platelet levels decreased in patients after exposure to PNT2258 with nadir at approximately 9 days post-onset of infusion. The decrease was generally ≤grade 1 and transient in nature, with spontaneous resolution without the need for transfusion or other supportive measures and without evidence of bleeding diathesis.

## Discussion

Progress in cancer therapeutics requires development and evaluation of novel strategies for drug delivery and interference of tumor cell signaling. One such novel strategy is the delivery of oligonucleotides into tumors in order to influence regulation of the cancer genome. PNT2258 is a first-in-class agent where a native unmodified DNA (24-base) oligonucleotide is delivered to a nuclear genomic target via a protective liposomal nanoparticle and the first to target the regulatory upstream region of the BCL2 gene in this manner. Although other oligonucleotide constructs have been proposed and evaluated in clinic (although none targeting nuclear DNA), successful systemic delivery has been an issue and the complex chemical nature of these constructs has negatively influenced their clinical toxicity profile [7, 23]. DNA oligonucleotide-mediated suppression of genomic DNA (i.e., prior to transcription) could provide a more efficient and less toxic means of down-regulating oncogene expression and promotion of tumor killing when compared to standard cytotoxic therapy, post-translational targeting of mRNA (via RNA-based oligonucleotides), or the inhibition of proteins (via small molecule protein inhibitors) [7, 24–36]. Further, inhibition of proteins by small molecule inhibitors has resulted in “off-target” toxicity, as has been the case with other agents, including previously tested BCL2-targeted therapeutics [27, 29, 32, 34–42].

PNT2258, and other related DNAi molecules, allows for clinical examination of whether targeting the upstream “regulatory” areas of the tumor genome with DNA oligonucleotides could be safe and tolerable and result in clinical activity without the characteristic toxicities of RNA-based oligonucleotides, cytotoxic, and small molecule protein inhibitor therapeutic approaches [43, 44].

This phase 1 study of PNT2258 allows for the following conclusions: (1) PNT2258 administered intravenously on days 1 through 5 of a 21-day cycle is well tolerated through doses of 150 mg/m^2^. (2) PNT2258 has an acceptable safety profile without evidence of thrombocytopenia or neutropenia at the doses tested. (3) Pharmacologic studies confirmed that doses >32 mg/m^2^ resulted in human AUC levels above that required for anti-tumor effect in preclinical xenograft studies of BCL2-dependent tumors. (4) Human exposure levels exceeded that seen with other oligonucleotide drugs by 10–100-fold [45]. (5) PNT2258 administration resulted in decreases in lymphocyte and platelets counts, and (6) PNT2258 administration resulted in systemic delivery and was not restricted to the liver. Lastly, disease control was noted in 27 % (6 of 22) patients receiving PNT2258 as indicated by >2 cycles of disease stabilization in this treatment-refractory population.

Preclinical results using human tumor xenograft-bearing mice confirmed robust single-agent anti-BCL2 response in the WSU-DLCL model, a NHL line with *t*(14,18) [13]. Given the relatively wide dose range between the corresponding minimally effective exposure in the xenograft model (i.e., the 32 mg/m^2^ dose level in humans) and the maximum tested dose of 150 mg/m^2^, it suggests that additional PNT2258 doses and schedules could be explored in addition to the one reported here. There were no clinically unmanageable grade 3 or 4 toxicities, even in this heavily pre-treated population at any dose level tested.

This intravenously administered agent displayed favorable pharmacologic features with relatively long (i.e., 1–2 day) half-life consistent with the goal of maximum exposure of the DNA-containing liposome nanoparticle to all tissues in the body and without evidence of cumulative toxicity secondary to the 5-day infusion schedule or evidence of significant hepatic sequestration.

While patients entering this pilot phase 1 trial of PNT2258 were not subjected to biopsy and therefore not preselected on the basis of their BCL2 expression status, the results of this trial are encouraging and indicate a need for further exploration of PNT2258 in patients diagnosed with BCL2-dependent tumors. On the basis of the results of this study, a phase 2 study (NCT01733238) has been initiated to explore the utility of PNT2258 as a single agent in patients with relapsed or refractory NHL.
